# Tissue-Specific Transcriptome Analysis Reveals Multiple Responses to Salt Stress in *Populus euphratica* Seedlings

**DOI:** 10.3390/genes8120372

**Published:** 2017-12-08

**Authors:** Le Yu, Jianchao Ma, Zhimin Niu, Xiaotao Bai, Wenli Lei, Xuemin Shao, Ningning Chen, Fangfang Zhou, Dongshi Wan

**Affiliations:** State Key Laboratory of Grassland Agro-Ecosystem, School of Life Sciences, Lanzhou University, Lanzhou 730000, China; yul15@lzu.edu.cn (L.Y.); majch15@lzu.edu.cn (J.M.); niuzhm16@lzu.edu.cn (Z.N.); bxt15138691581@163.com (X.B.); leiwl15@lzu.edu.cn (W.L.); shaoxm15@lzu.edu.cn (X.S.); chennn2015@lzu.edu.cn (N.C.); zhouff16@lzu.edu.cn (F.Z.)

**Keywords:** *Populus euphratica*, salinity stress, transcriptome, differentially expressed gene, tissue-specific

## Abstract

Salt stress is one of the most crucial factors impacting plant growth, development and reproduction. However, information regarding differences in tissue-specific gene expression patterns, which may improve a plant’s tolerance to salt stress, is limited. Here, we investigated the gene expression patterns in tissues of *Populus euphratica* Oliv. seedlings using RNA sequencing (RNA-Seq) technology. A total of 109.3 million, 125bp paired-end clean reads were generated, and 6428, 4797, 2335 and 3358 differentially expressed genes (DEGs) were identified in leaf, phloem, xylem and root tissues, respectively. While the tissue-specific DEGs under salt stress had diverse functions, “membrane transporter activity” was the most significant leaf function, whereas “oxidation–reduction process” was the most significant function in root tissue. Further analysis of the tissue-specific DEGs showed that the expression patterns or functions of gene families, such as *SOS*, *NHX*, *GolS*, *GPX*, *APX*, *RBOHF* and *CBL*, were diverse, suggesting that calcium signaling, reactive oxygen species (ROS) and salt overly sensitive (SOS) pathways are all involved in ionic homeostasis in tissues from *P. euphratica* seedlings. The DEGs, for example the up-regulated antioxidant genes, contribute to ROS-scavenging induced by salt stress but result in decreased Na^+^ concentrations in root vasculature cells and in xylem sap, while the down-regulated *rbohF* leads to the reverse results. These results suggest that the divergence of DEGs expression patterns contribute to maintenance of ionic and ROS homeostasis in tissues and improve plant salinity tolerance. We comprehensively analyzed the response of *P. euphratica* seedlings to salt stress and provide helpful genetic resources for studying plant-abiotic stress interactions.

## 1. Introduction

Salinity is one of the most important environmental factors that limit plant growth, development and productivity [1]. Globally, almost 20% of the world’s cultivated land and nearly 50% of irrigated land suffer from saline stress [2]. Saline stress has far-reaching implications for economic sustainability, food security and the irreplaceable biodiversity of the affected areas [3]. Therefore, a comprehensive understanding of the molecular and physiological mechanisms of salt tolerance in plants will not only help us uncover plant adaptive molecular mechanisms but also contribute to the cultivation of plants/crops that can tolerate high salt stress [4].

Plants are damaged by high levels of sodium ions, which lead to inhibited plant growth and cause cell death and enormous losses in yield [5]. To survive under salt stress, plants have to rapidly sense and respond to stress using the following three strategies: (1) reduction of cell expansion in root tips and stomatal closure in young leaves; (2) exclusion of Na^+^ by the roots and decreased accumulation of Na^+^ in the leaves; and (3) compartmentalization of Na^+^, K^+^ and Cl^−^ ions in tissue vacuoles and transportation to different tissues [6]. A series of signaling and regulatory pathways mediated by plant hormones, specific transcription factors and functional proteins are required for the osmoregulation, cell protection and acclimation that support these strategies in plants [6,7,8,9,10,11]. For example, three gene families are involved in the process of ion uptake in plant roots [12]. In the salt overly sensitive (SOS) pathway, the SOS3–SOS2 complex senses salt signals and further activates SOS1, which has a role in loading Na^+^ into xylem via phosphorylation [13]. In the K^+^/H^+^ antiporter (NHX) family of proteins, multiple gene copies in plants have different specificities for different ions and regulate K^+^, Na^+^ and H^+^ homeostasis in intra-cellular compartments [14,15]. High-affinity K^+^ transporters (HKT) can transport excess Na^+^ from the roots to avoid damaging the leaves [16,17]. Interestingly, reactive oxygen species (ROS), which are an important signaling molecule, are involved in most salt response processes. In *Populus euphratica* Oliv., salt stress induces H_2_O_2_ production and enhances the Na^+^/H^+^ exchange [18], while plasma membrane (PM) H^+^-ATPase enhances H^+^ afflux and decreases the pH in apoplast, leading to an H_2_O_2_ burst. The excessive H_2_O_2_ subsequently stimulates Na^+^/H^+^ antiport to regulate ion homeostasis via Ca^2+^-SOS pathway [18,19,20]. Moreover, plant hormones, such as abscisic acid (ABA) [21] and cytokinin [22], also participate in salt stress tolerance in plants. These responses have been observed in both herbs and trees through the characterization of genes activated in response to abiotic stress [23,24,25,26]. Furthermore, a number of transcription factors (TFs) in plants activate a spectrum of defenses against salt stress [27]. Nevertheless, studies on how plants resist salt stress via ion compartmentalization in tissue vacuoles and ion transportation to different tissues are limited, especially in wood plants. Changes in the gene expression patterns between different plant tissues confronted with salt stress may provide comprehensive insights into the genetic basis of plant response to salt stress.

*P. euphratica* is a phreatophyte distributed in arid or semi-arid regions. Because of its ability to tolerate salt [28], *P. euphratica* is considered a valuable natural genetic resource for the breeding of salt tolerance in plants. *P. euphratica* has evolved multiple morphological traits to adapt to environmental salinity, such as a specific hydraulic system and succulent leaves [28], which result in salt dilution and further reduce salt damage to tissues. *P. euphratica* can grow in the presence of 200 mM NaCl and even survives in 400 mM NaCl [29]. Salt uptake is compartmentalized in the apoplast and vacuole [30,31], which contribute to balancing Na^+^ uptake and maintaining a favorable Na^+^/K^+^ ratio in *P. euphratica* cells. Previous studies have indicated that many genes are involved in these processes. Na^+^/H^+^ antiporters (e.g., SOS1, NHD2 and NaHD1) and H^+^ pumps (e.g., PM H^+^-ATPase) in *P. euphratica* involved in ion uptake and transportation are up-regulated under salt stress [32,33]. The H^+^-ATPase and HKT families in *P. euphratica* have expanded and produce more copies than in the *Populus trichocarpa* genome [34]. The expansion of gene families and the enhanced activities of these ion-transporting proteins may provide a key molecular basis for the improved tolerance of *P. euphratica* to salinity. It is obvious that improving the salt tolerance of *P. euphratica* occurs not only through molecular regulation but also through physiological and anatomical adjustments. Therefore, to investigate global plant response to salt stress, tissue-specific responses are necessary in *P. euphratica*. The transcriptome profiles from *P. euphratica* based on next-generation sequencing, make it possible to uncover comprehensive gene expression information from different tissues which could contribute to elucidating the different mechanisms used by different tissues when confronted with salt stress. Unfortunately, these types of studies are limited, especially in trees.

Here, we used transcriptome profiling to investigate the effect of salt stress on tissues from *P. euphratica* seedlings. The transcriptomes of four tissues from *P. euphratica* seedlings grown with 0 mM, 150 mM or 300 mM NaCl were compared to identify DEGs (differential expression genes) in response to salt stress. The acquired data and the results provided a clear pattern of the comparative gene expression in response to salt stress.

## 2. Materials and Methods

### 2.1. Plant Materials and RNA Extraction

Two-year-old *P. euphratica* seedlings were collected from Akesu, Xinjiang province, China and planted in pots with loam soil. All seedlings were grown in a greenhouse with a 16 h/8 h day/night photoperiod (6:30–22:30) and 60% humidity. The seedlings used for the experiment were cultivated and treated with a solution containing either 0 mM, 150 mM or 300 mM NaCl for 7 days. For RNA sequencing (RNA-Seq), total RNA was extracted from leaf, phloem, xylem and root tissues from each seedling using the CTAB procedure [35]. Three replicates from three individual seedlings were treated with the same salt concentration. Each tissue from a total of 36 samples was collected at similar stages. The integrity and quality of the RNA samples were examined with a NanoDrop 8000 UV–Vis Spectrophotometer (Thermo, Darmstadt, Germany). The A260/A280 ratio of the RNA samples was between 1.9 and 2.1, and the RNA integrity number (RIN) values ranged from 8.6 to 10.0.

### 2.2. Illumina Sequencing

The preparation of whole transcriptome libraries and the sequencing were performed by the Annoroad Gene Technology Corporation (Beijing, China). Whole transcriptome libraries were constructed using the New England Biolabs Next, Ultra Directional RNA Library Prep Kit for Illumina (New England Biolabs, Ipswich, MA, USA), in accordance with the manufacturer’s instructions. The libraries were controlled for quality and quantified using a BioAnalyzer 2100 system and quantitative PCR (qPCR) (Kapa Biosystems, Woburn, MA, USA). The resulting libraries were initially sequenced on a HiSeq 2500 instrument (Illumina, San Diego, CA, USA) and paired-end, 125 nucleotide reads were generated. The sequencing data were submitted to the NCBI Sequence Read Archive. The project accession number is SRP116293. The accession numbers for the complementary DNA (cDNA) libraries obtained from the controls and the salt-stressed leaf, phloem, xylem and root samples are SRX3139499, SRX3139976, SRX3139977 and SRX3140050, respectively.

### 2.3. Raw Sequence Processing and Read Mapping

We first cleaned the raw sequences by removing adaptor sequences, empty reads, low quality sequences (reads with too many ‘N’s) and reads with more than 10% Q <20 bases. The cleaned reads from each library were used for later analyses. The RNA-Seq reads from all the samples were mapped onto the reference genome [34] using Bowtie2 (version 2.2.5) [36] and Tophat2 (version 2.1.0) [37] software with default parameters, to determine the expression levels of the identified genes. Fragments per kilo-base of gene per million mapped fragments (FPKM) values from each gene were calculated using Cufflinks software (version 2.2.1) [38]. Genes with a summed FPKM <1 from all 36 samples were filtered out, and the rest of the genes were used for further analyses.

### 2.4. Identification of Differential Expression Genes

To identify the DEGs between the salt-stressed and control samples and between the samples collected from the different salt concentrations, we merged all the transcripts using Cuffmerge and then identified the differentially expressed genes between the pairs of samples from each tissue type using Cuffdiff. DEGs were identified by applying a cutoff *p*-value < 0.05 (false discovery rate, FDR Bonferroni corrected); DEGs with a fold-change >2 were defined as up-regulated and those with a fold-change <0.5 were defined as down-regulated. Tissue-specific DEGs were identified by filtering DEGs that were only present in one tissue type.

### 2.5. Clustering Analyses

*K*-means clustering of the transcript expression patterns was based on FPKM values, using the *K*-Means/*K*-Medians Support MultiExperiment Viewer 4.9 (http://www.tm4.org/mev). FPKM values for each transcript were normalized and then zero-centered by subtracting the median of the 12 values from each sample. The optimal number of clusters was defined by the figure of merit (FOM) application [39] within MEV4.9. The *K*-means analysis was performed using Euclidean distances. 

### 2.6. GO and KEGG Enrichment Analyses

Gene Ontology (GO) functional classification for all the genes found in UniGene was performed using the Web Gene Ontology Annotation Plot software (WEGO; http://wego.genomics.org.cn). The classifications were counted using custom-designed Perl scripts. Kyoto Encyclopedia of Genes and Genomes (KEGG) enrichment was carried out using KOBAS 3.0 software (http://kobas.cbi.pku.edu.cn/anno_iden.php) with homologous genes identified from *P. trichocarpa* using a reciprocal Basic Local Alignment Search Tool (BLAST) analysis [40,41].

### 2.7. DEG Network Analysis

The network was constructed using Cytoscape V3.5.0 with the EnrichmentMap Plug-in [42]. Significant terms of clusters were selected to construct options using the default parameters (FDR < 0.01).

### 2.8. Quantitative Real-Time PCR 

To verify the reliability of the RNA-Seq analyses, six candidate DEGs were selected for quantitative Real-Time PCR (qRT-PCR) analysis. A portion (0.5 µg) of DNase I-treated total RNA from 36 samples was transformed into single-stranded cDNA with a PrimeScript 1st Strand cDNA Synthesis kit (TaKaRa, Dalian, China). The cDNA templates were diluted 20-fold and amplified with a CFX96 Real-Time PCR detection system (Bio-Rad, Singapore) and SYBR Premix ExTaq™ (Takara). The templates were amplified using the following program: 95 °C for 15 s, 60 °C for 30 s and finally 72 °C for 20 s. The primers were designed using the Primer Premier 5 software (PREMIER Biosoft, Palo Alto, CA, USA) and are listed in Supplementary File S1. Three biological replicates were used for each gene. The relative expression levels of the DEGs, which were normalized to the expression of the internal reference gene actin, were calculated according to the 2^−ΔΔct^ method [43].

## 3. Results and Discussion

Transcriptional analysis of the callus tissues from *P. euphratica* under salt stress showed a series of tolerance mechanisms [44,45]. However, as a model wood plant species, *P. euphratica* displays higher tolerance to adverse environmental conditions and diversified adaptive phenotypes, such as leaf polymorphisms, leaf succulence, root geotropism and dense wood structure [46,47]. These polymorphism features provide more plastic strategies for *P. euphratica* to survive in severe conditions. Furthermore, as a high salt tolerant species, the response of *P. euphratica*, as a high salt-tolerant species, to salt stress may be different under moderate and severe salt stresses. Therefore, we compared the transcriptomic changes in four tissues from *P. euphratica* seedlings exposed to different salt concentrations. A global view of the gene expression profiles and a large-scale tissue-specific transcriptome profile of *P. euphratica* based on deep sequencing technology provided more accurate insights into the genes that responded to high salinity.

### 3.1. RNA-Seq and Mapping P. euphratica Sequences

**Figure 1:**
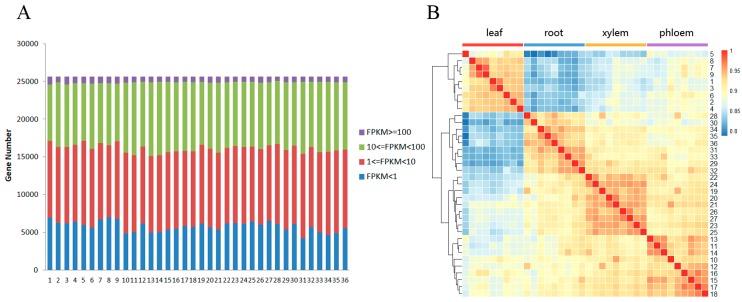
Effect of salt stress on the *Populus euphratica* transcriptome. (**A**) Number of genes expressed in the treated samples. (**B**) Cluster heat map showing the global relationships of the expressed genes between samples. The heat map was made using the default settings and cor and hclust functions in R. 1-36 stand for the 12 samples with three repeats sequenced for leaf, phloem, xylem and root. FPKM: Fragments Per Kilo-base of gene per Million mapped fragments.

The libraries and pair-end sequenced reads from the Illumina HiSeq 2500 experiments were constructed and sequenced to comprehensively understand the global profile of the plant’s transcriptome in response to salt stress (Supplementary File S2). Next, the raw reads were filtered and mapped to the reference *P. euphratica* genome using Bowtie2 software [36], which also obtained the gene FPKM values. In total, we generated 109.3 million 125bp, paired-end clean reads. On average, 89.5% of the clean reads were mapped to the reference genome (Supplementary File S3). To reduce the effects of background transcription, only those genes with a summed FPKM > 1 from all the samples were retained; thus 25,604 genes were used for further analyses. Of these genes, approximately 70% had FPKM values in the range from 1 to 100. High expression values (FPKM ≥ 100) accounted for approximately 3% of the total filtered genes, and approximately 23% of the expressed genes had low expression values (FPKM < 1) (Figure 1A). The correlation heat map presented in Figure 1B illustrates that samples from the same tissue type were clustered together, and all the correlation coefficients between pairs were over 0.8, indicating that the tissue-specific gene expression patterns in *P. euphratica* under salt stress were diverse (Figure 1B).

### 3.2. Identification of DEGs from Plants Grown under Different Salt Concentrations

**Figure 2:**
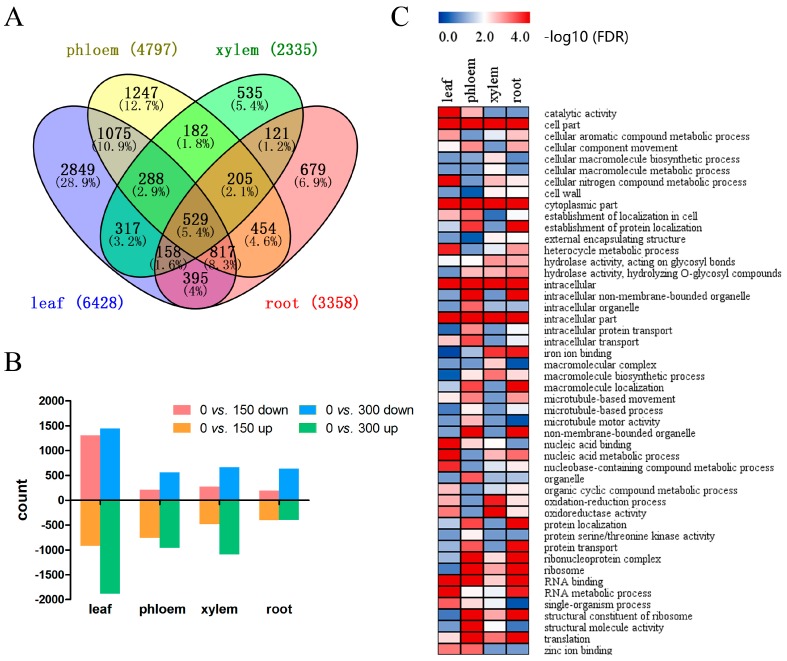
(**A**) Venn diagram showing the unique and shared DEGs between the transcriptomes from the treated and control *P. euphratica* samples; (**B**) Up-regulated and down-regulated differentially expressed genes (DEGs) from the 0 vs. 150 mM and 0 vs. 300 mM comparisons in four tissues; (**C**) Gene ontology (GO) enrichment heat map for all the DEGs within the different tissues. FDR: false discovery rate.

To identify the global transcriptional changes between in tissues, we compared the samples in pairs within each tissue. The DEGs identified from 0 vs. 150 mM, 0 vs. 300 mM and 150 mM vs. 300 mM in each tissue were merged and are shown in a Venn diagram (Figure 2A). The read counts were compared between treatments within tissues to identify significant DEGs using a *p*-value <0.05 (FDR Bonferroni corrected) as a cutoff value. In total, 9851 DEGs were identified, 6428 in leaf tissue, 4797 in phloem tissue, 2335 in xylem tissue and 3358 in root tissue. The expression patterns and annotations for the DEGs are listed in Supplementary File S4. DEGs with a fold-change >2 were considered up-regulated, while DEGs with a fold-change <0.5 were considered down-regulated. Genes with up- or down-regulation between the control, 150 NaCl mM and 300 mM NaCl groups in different tissues are displayed in Figure 2B. In plants grown with 150 mM NaCl, 1300, 206, 274 and 193 DEGs were up-regulated, while 919, 761, 479 and 401 DEGs were down-regulated in the leaf, phloem, xylem and root tissues, respectively. Meanwhile, in the leaf, phloem, xylem and root tissues of plants grown with 300 mM NaCl, 1444, 562, 664 and 634 DEGs were up-regulated, and 1883, 961, 1091 and 396 DEGs were down-regulated, respectively. The number of DEGs in all the tissues mostly increased when the salt concentration increased from 150 to 300 mM NaCl, indicating that salt-tolerant genes present different expression patterns when confronted with different levels of salt stress. GO enrichment for the DEGs in the four tissues was also performed (Figure 2C, Supplementary File S5). The functions of the DEGs in the leaf tissues were mainly enriched for “cell part”, “intracellular” and “RNA metabolic process”, suggesting that high sodium concentrations accumulated in the leaves because of apoplastic accumulation, probably leading to changes in the cell wall properties that allowed for enhanced cation binding [31]. In contrast, “cytoplasmic part”, “intracellular” and “ribonucleoprotein complex” were the most significant terms in the root tissue samples. These results showed that DEGs have diverse functions in different tissues.

### 3.3. The Same Gene Functions and Expression Patterns Were Observed in Response to Salt Stress in Different Tissues

**Figure 3:**
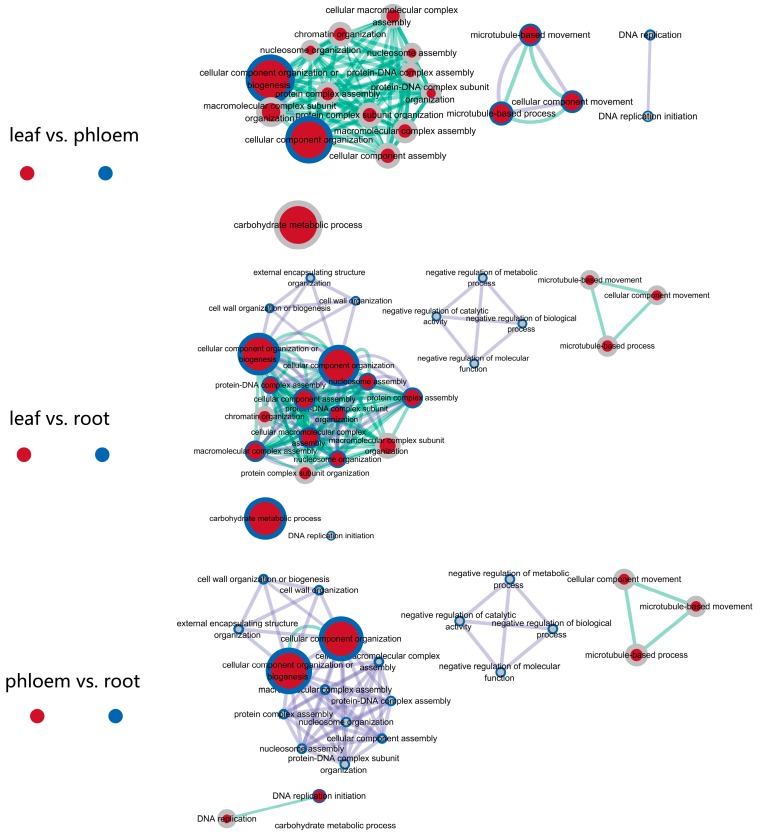
Network analyses for the DEGs with similar expression patterns in *P. euphratica* leaf, phloem, xylem and root tissues. GO modules for the biological processes were visualized using EnrichmentMap in Cytoscape [42]. Nodes and their periphery represent different gene sets, and the node size represents how many genes are in the gene set. Edges represent mutual overlap.

To further explore the diverse functions of the tissues under salt stress and to elucidate the dynamic changes in the transcriptomes from different samples, we grouped the DEGs within each tissue using a *K*-Means clustering algorithm. We grouped seven, six, seven and seven clusters in the leaf, phloem, xylem and root tissues, respectively (Supplementary File S6). Some of these clusters in different tissues shared the same expression patterns, including similar expression trends, suggesting that these genes may participate in similar biological processes under salt stress in different tissues. For example, cluster 2 from leaf tissue (1690 in 6428) (Figure S1), cluster 2 from phloem tissue (1078 in 4797) (Figure S2), and cluster 1 from root tissue (669 in 3358) (Figure S4) are the biggest clusters that showed the same expression patterns; these clusters were all up-regulated in plants exposed to 150 mM NaCl and down-regulated in plants exposed to 300 mM NaCl. Cluster analysis showed that there is no such a cluster in xylem tissues, which may be because of the insufficient total number of DEGs in xylem. Because few data points from xylem tissue agreed with the criteria, we ignored the clusters from xylem tissue. Significant GO terms for these three clusters are presented in Supplementary File S7. GO enrichment analyses showed that some of the enriched GO terms were shared among these three clusters. We further constructed network analyses with the enriched GO terms from two different tissues, as shown in Figure 3.

In the leaf vs. phloem, leaf vs. root and phloem vs. root biological process regulatory networks, the “cellular component organization or biogenesis” functions were the most significant terms in the leaf, phloem and root tissues, indicating that various cellular components in the whole plant may be greatly affected by salt stress. The GO terms from the leaf and root tissue samples were enriched for the same types of cellular processes, including “microtubule-based movement”, “microtubule-based process” and “cellular component movement”, while samples from the phloem and root tissues only in had “DNA replication initiation” in common. Na^+^ can be transported to the shoots by the rapidly moving transpiration stream in the xylem and can be returned to the roots via the phloem [48]. This observation suggests that microtubule-based processes play an important role in the transport of Na^+^ from the shoots to the roots. Additionally, samples from leaf and root tissues had the most biological processes in common, such as “protein–DNA complex assembly”, “nucleosome assembly” and “protein complex assembly”, indicating that these two tissues may have genes with synergistic functions in regulating ion homeostasis (Figure 3). GO enrichment analyses in the different tissues showed that several GO terms were same, which indicated that there may be DEGs with similar functions. For example, the *AFH1* and *NRP2* genes showed similar functions, but were differentially expressed in the phloem and root. These results indicate that different tissues can respond to salt stress by the same means through the same gene expression patterns.

### 3.4. Tissue-Specific DEGs in P. euphratica under Salt Stress

**Figure 4:**
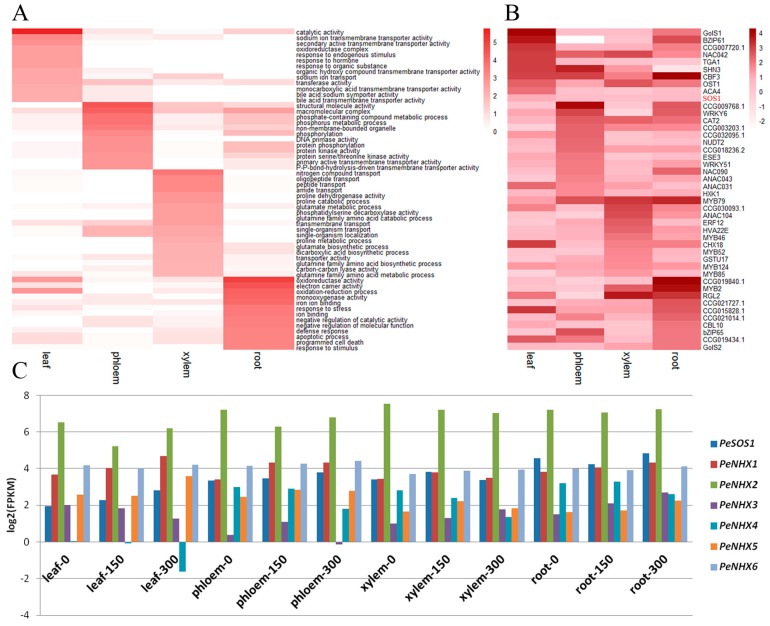
Analysis of tissue-specific DEGs. (**A**) Heat map showing log_10_ (*p*-value) values of the most significant GO terms related to salt-tolerance; (**B**) Heat map showing log_2_ (fold change) values of DEGs related to salt-tolerance; (**C**) Expression patterns for the *PeSOS1* and *PeNHX* gene families.

Tissue-specific genes are a series of genes whose expression and function are preferred in one or many tissues, and identification of tissue-specific genes is helpful to better understand the relationships among genes in different tissues [49]. In this study, a total of 2849, 1247, 535 and 679 tissue-specific, differentially expressed genes were found in the leaf, phloem, xylem and root tissues, respectively (Figure 2A). GO enrichment analyses showed that tissue-specific DEGs have diverse functions. As shown in Figure 4A, “catalytic activity”, “sodium ion transmembrane transporter activity” and “secondary active transmembrane transporter activity” were the most significant functions of the leaf-specific DEGs; “structural molecule activity”, “macromolecular complex” and “phosphate-containing compound metabolic process” were the most significant functions of the phloem-specific DEGs; “nitrogen compound transport”, “oligopeptide transport” and “peptide transport” were the most significant functions of the xylem-specific DEGs; and “oxidoreductase activity”, “electron carrier activity” and “oxidation-reduction process” were the most significant functions of the root-specific DEGs. These enriched GO terms indicated that the leaf tissues responded to salt stress mainly via membrane transporter activities, while the root tissue responded to salt stress mainly through oxidation-reduction processes.

The ability of plants to manage salt stress relies on the root system and is based on the capacity to control ion uptake in root cells, xylem loading and salt removal from the xylem and upper tissues. Plant root cells generally absorb Na^+^ from the soil through different channels. Channel permeation and apoplasts are the main pathways for Na^+^ influx under salinity stress (Figure S6). In the salt overly sensitive (SOS) pathway, Na^+^ crosses the plasma membrane to the apoplast or soil solution and *NHX1* (Na^+^-H^+^ exchanger) partitions Na^+^ within the vacuole, jointly regulates the cytosol Na^+^ concentrations and plays a vital role in response to salt stress; thus, *SOS* and *NHXs* are the core genes that have been proposed to regulate cellular signaling under salt stress and maintain ion homeostasis in cells [50,51]. Here, we investigated the expression pattern of these genes; their log_2_ values (fold change) are shown in Figure 4B. *PeSOS1*, which is mainly expressed in leaf tissue (Figure 4B), showed significant up-regulation (Figure 4C). *PeSOS1* was slightly down-regulated in phloem from plants treated with 300 mM NaCl and in the root tissue with 150 mM NaCl treatment, indicating that *PeSOS1* can extrude Na^+^ into the soil and accumulate Na^+^ ions that are transported long-distance to the leaves by the transpiration stream [52]. The NHX family of vacuolar Na^+^/H^+^ antiporters exhibit multiple transport Na^+^/H^+^ activities [53,54]. In *P. euphratica*, the NHX family comprises six NHX members encoded by *PeNHX1-6* genes. Among them, *PeNHX1*, *PeNHX2* and *PeNHX5* are expressed in leaf tissues, indicating a leaf-specific function of these genes; *PeNHX1/5* showed continuous up-regulation except in xylem tissues from plants treated with 300 mM NaCl. Furthermore, *PeNHX2*, *PeNHX3*, *PeNHX4* and *PeNHX6* exhibited prominent, differential expression patterns (Figure 4C). Briefly, the *PeNHXs* have diversified not only in their gene expression patterns, but also in their Na^+^/H^+^ transport capacities. *PeNHX1/2/5* mediates greater Na^+^ tolerance than *PeNHX3/4/6* [55], suggesting that diversity among the *PeNHX* in expression patterns and Na^+^/H^+^ transport capacities improve *P. euphratica* by regulating Na^+^ transport across different tissues. In conjunction with other genes’ expression, such as HKTs (high affinity K^+^ transporters), an expanded gene family in *P. euphratica* genome [34], whose expression was up-regulated in the root and leaf, could contribute to maintaining ion homeostasis [16,17]. Taken together, our results further verified that plants decrease the Na^+^ concentration in the transpiration stream and increase salt tolerance via the following two patterns: (1) Na^+^ retrieval in the root by directly unloading sodium from the xylem sap to xylem parenchyma cells and (2) Na^+^ recirculation in the shoot through the removal of Na^+^ from the xylem sap and then transporting Na^+^ from phloem companion cells into the phloem sieves [56]. These processes involve a number of genes in various pathways. For example, shoot Na^+^ homeostasis in plants grown in saline soils is conferred by reactive oxygen species (ROS) regulation of xylem-sap Na^+^ concentrations. In *P. euphratica*, Na^+^/H^+^ exchange could be enhanced by H_2_O_2_ production induced by salt stress [18], while PM H^+^-ATPase enhanced H^+^ afflux and decreased the pH in the apoplast, leading to H_2_O_2_ burst. The excessive H_2_O_2_ subsequently stimulates Na^+^/H^+^ antiport via Ca^2+^-SOS pathway to regulate ion homeostasis among tissues [18,19,20]. Lack of *rbohF*-dependent salinity-induced vascular ROS accumulation leads to increased Na^+^ concentrations in root vasculature cells and xylem sap, causing delivery of Na^+^ to the shoot [57]. A previous study indicated that lack of *rbohF* function promotes the accumulation of more shoot Na^+^ than in controls [58]. Our results showed that *rbohF* in *P. euphratica* was up-regulated in the four tissues under both 150 mM and 300 mM NaCl stresses, especially in xylem tissues, suggesting that salt tress induces *rbohF* expression, which decreases ROS accumulation in vascular cells and Na^+^ accumulation in shoots. The increase in stele ROS accumulation led to reduced net Na^+^ influx in roots, decreased Na^+^ xylem loading and to root K^+^ retention and subsequent enhanced salinity tolerance [59]. However, excessive ROS accumulation induced by salt stress is also a threat that can damage cell membranes and promote proteins redox [60]. Interestingly, antioxidant systems can maintain the ROS balance in plant cells. In this study, *APX2* (ascorbate peroxidase) and *GPX4* (guaiacol peroxidase) were up-regulated in the four tissues, but *GR* (glutathione reductase) expression was induced only by 300 mM NaCl stress in root. The improved expression of antioxidant enzymes can detoxify elevated ROS levels [61]. Therefore, the up-regulation of antioxidants defenses contributes to halophyte species adaptation in salinity stress [18].

Multiple expression patterns in different tissues may present a novel cognition of these salt-tolerant genes. The expression levels of galactinol synthase (*GOLS*) genes, such as *GolS1* and *GolS2*, increased in the leaves and the roots (Figure 4B), confirming that compatible solute formation is also an important mechanism used by *P. euphratica* plants to cope with salt stress [62]. The expression of calcineurin B-like (*CBL10*) increased in the roots, indicating that calcium (Ca^2+^) signaling, which activates Na^+^⁄H^+^ antiporters by triggering a signaling cascade, is involved in salt tolerance in *P. euphratica* [63,64]. These data suggest that salt stimuli significantly enhanced Ca^2+^ accumulation in *P. euphratica* roots, subsequently increasing Ca^2+^ levels in the cytosol, which mediates K^+^⁄Na^+^ homeostasis in cells and improves salt stress tolerance [19,20].

### 3.5. Full-Tissue DEGs in Response to Salt Stress: Diversified Expression Patterns in Different Tissues

In total, we identified 529 DEGs in all four tissue types (Figure 2A and Figure S5). GO enrichment illustrated that these DEGs were highly enriched in processes associated with “cytoplasmic part”, “antioxidant activity” and “xyloglucan: xyloglucosyl transferase activity”. KEGG enrichment results showed that terms related to “plant hormone signal transduction”, “pentose and glucuronate interconversions” and “carotenoid biosynthesis” were the most significant pathways (Figure S5 and Supplementary File S8). There were 23 DEGs with similar expression patterns, while the rest had diverse expression patterns in the four tissues types examined. For example, low temperature and salt-responsive protein *RCI2A* and the *ABF2* and *ABF3* genes, showed significant up-regulation in all four tissues as the salt concentration increased, indicating that these genes may play a vital role throughout the plant while it is confronting salt stress. The No Apical Meristem (NAC) domain transcriptional regulator superfamily protein, *ANAC017*, was down-regulated under moderate salt stress but up-regulated in all four tissues under severe salt stress, showing a dose-dependent response. These genes may function as whole-plant salt-tolerant genes in *P. euphratica*; thus identifying their detailed functions requires further analysis.

### 3.6. Transcription Factors in P. euphratica Involved in Response to Salinity Stress

Distinct TF family genes mediate a plant’s adaptation to salt stress [65]. Of the 9851 DEGs identified in this study, 887 differentially expressed transcription factors were identified under salt stress. Numerous transcription factors, including 98 MYBs, 96 ERFs and 60 NACs, which are key regulators of plant responses to abiotic stresses, were identified as differentially expressed (Supplementary File S9). Of these, the expressions of 223 TFs were leaf-specific, 111 TFs were phloem-specific, 61 TFs were xylem-specific, and 71 TFs were root-specific. MYB, bHLH and NAC were the top three leaf-specific TF families, while MYB, bHLH and Dof were phloem-specific TF families. The MYB, ERF and C2H2 gene families were xylem-specific, whereas the GRAS, ERF and bHLH gene families were root-specific (Supplementary File S10). Of the differentially expressed transcription factors, the *WRKY* family of TFs has expanded and includes ten species-specific *WRKY* genes through tandem or segmental duplication in the *P. euphratica* genome compared with its sister species, *P. trichocarpa* [46]. The formation and alteration of *WRKY6* with new gene expression features may have improved the high salinity tolerance in *P. euphratica* (Figure 4B). Furthermore, increased TF expression enhanced salt stress tolerance by regulating other gene expressions. For example, overexpressing *NAC* from *P. euphratica* significantly inhibits *AtHKT1* expression and decreases Na^+^/K^+^ ratios in the roots and leaves of *Arabidopsis* [52,66]. Here, *NAC42* expression increased in the leaf and xylem tissues, while *NAC90* expression increased in the phloem and root tissues (Figure 4B). The significant divergence in the expression patterns between the two *NAC* genes may help *P. euphratica* adapt to adverse environments. Of note, *MYB46* expression increased only in xylem tissue (Figure 4B), confirming that *MYB46* expression in *P. euphratica* is involved as a central and direct regulator of secondary wall component biosynthesis [67].

### 3.7. Validation of Gene Expression Patterns by qRT-PCR

**Figure 5:**
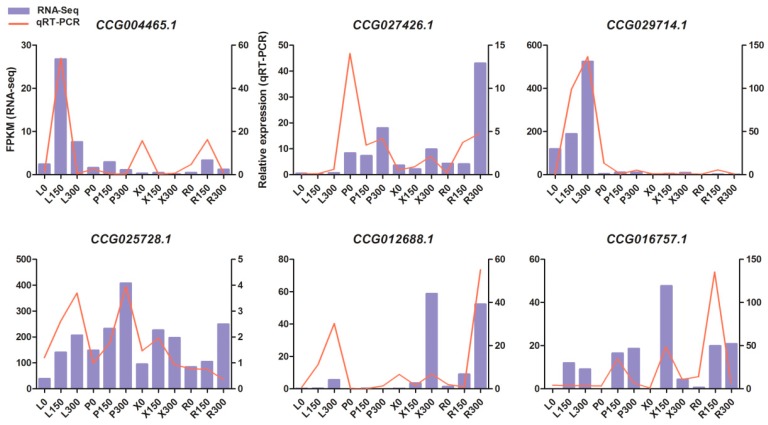
qRT-PCR (quantitative real-time PCR) verification of six selected DEGs. Comparison of RNA sequencing (RNA-Seq) data (blue bar) with qRT-PCR data (red line). The normalized expression levels (FPKM) from the RNA-Seq results are indicated on the y-axis to the left. The relative qRT-PCR expression level is shown on the *y*-axis to the right. Actin was used as an internal control. Both methods agree with each other in showing similar gene expression trends.

To validate the gene expression inferred from the RNA-Seq experiments, six candidate DEGs were selected for qRT-PCR analysis; their annotations in *Arabidopsis* are displayed in Supplementary File S11. The six candidate genes included plant ubx domain-containing protein 2 (*PUX2*), Su (var) 3-9-related protein 5 (*SUVR5*), *XERICO*, transcription factor IIIA (*TFIIIA*), bromodomain and ATPase domain-containing protein 1 (*BRAT1*) and one gene with no annotation. These genes had displayed divergence and significant expression patterns under salt stress in different tissues. For example, *CUVR5* (CCG029714.1) showed a sharp increase only in leaf with an increase in the salt concentration, indicating that this gene may play a leaf-specific role in confronting salt stress. Although the fold-changes in their expression detected by sequencing did not exactly match those detected by qRT-PCR, the detected expression patterns were mostly consistent for all the selected genes, confirming the reliability of the RNA-Seq results (Figure 5).

## 4. Conclusions

In this study, we explored tissue-specific transcriptomic changes and identified a large number of DEGs in response to salt stress in *P. euphratica*. Gene ontology and KEGG pathway analyses of the DEGs revealed the similarities and differences in the regulation profiles of the seedlings exposed to salt stress, which indicated global mechanisms for salt resistance in different tissues in *P. euphratica*. Moreover, the divergence of DEGs expression patterns, such as *SOS*, *NHX*, *GolS*, *GPX*, *APX*, *RBOHF* and *CBL* contribute to maintenance of ionic and ROS homeostasis in tissues and improve plant salinity tolerance. Additionally, the differential expression of transcription factors promoted complex crosstalk between response pathways among tissues under salt stress. These differences suggest that these genes may play crucial roles in *P. euphratica* salt-tolerance.

## Supplementary Materials

The following are available online at www.mdpi.com/2073-4425/8/12/372/s1. Figure S1: Clusters 1–7 for the 6428 DEGs in leaf tissue. Figure S2: Clusters 1–6 for the 4797 DEGs in phloem tissue. Figure S3: Clusters 1–7 for the 2335 DEGs in the xylem tissue. Figure S4: Clusters 1–7 for 3358 DEGs in the root tissue. Figure S5: Expression pattern and enrichment analyses of the DEGs in within the four tissues. Figure S6: A putative pathway related to ionic homeostasis. Figure S7: The expression patterns of genes involved in reactive oxygen species homeostasis. File S1. Gene primers for qRT-PCR. File S2. Output statistics from the Illumina-Solexa sequencing. File S3. Statistics for the clean reads and alignment rates in the 36 samples. File S4. Expression and annotation of DEGs in the four tissues. File S5. GO terms for all the DEGs. File S6. Clusters of DEGs in the four tissues. File S7. GO terms for each cluster in the four tissues. File S8. GO and KEGG terms for the common DEGs. File S9. Differentially expressed Transcription factors in *P. euphratica*. File S10. List of tissue-specific TFs. File S11. The expression levels (FPKM) from the RNA-seq experiments and the relative qRT-PCR expression levels for the six selected DEGs.
