# Supplementary material for: Tissue-Specific Transcriptome Analysis Reveals Multiple Responses to Salt Stress in Populus euphratica Seedlings
Source: Genes (Basel). 2017 Dec 8;8(12):372. doi: 10.3390/genes8120372 (PMC5748690; doi:10.3390/genes8120372)

**Supplementary File S6. clusters for DEGs in four tissues.**

**Figure S1. 7 cluster 1-7 for 6428 DEGs in leaf.**


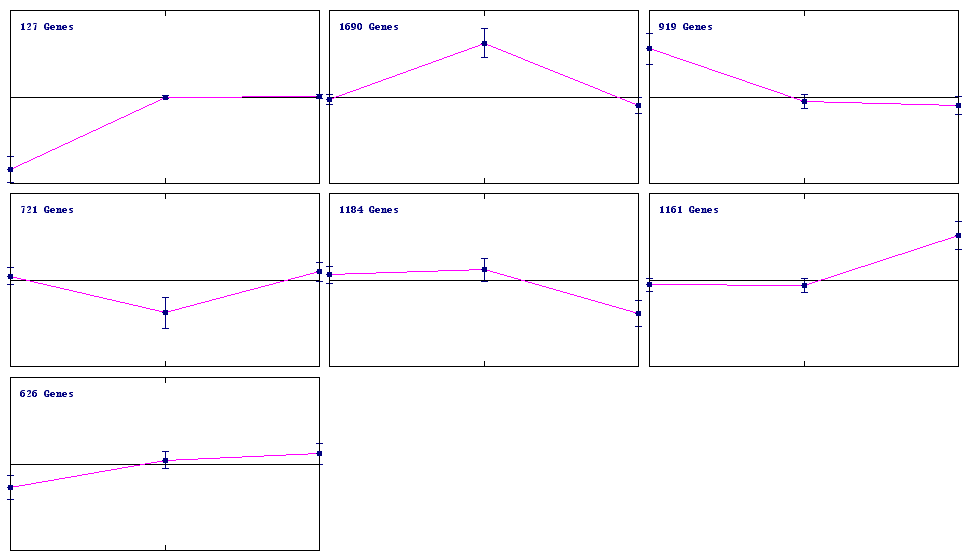


**Figure S2. 6 cluster 1-6 for 4797 DEGs in phloem.**


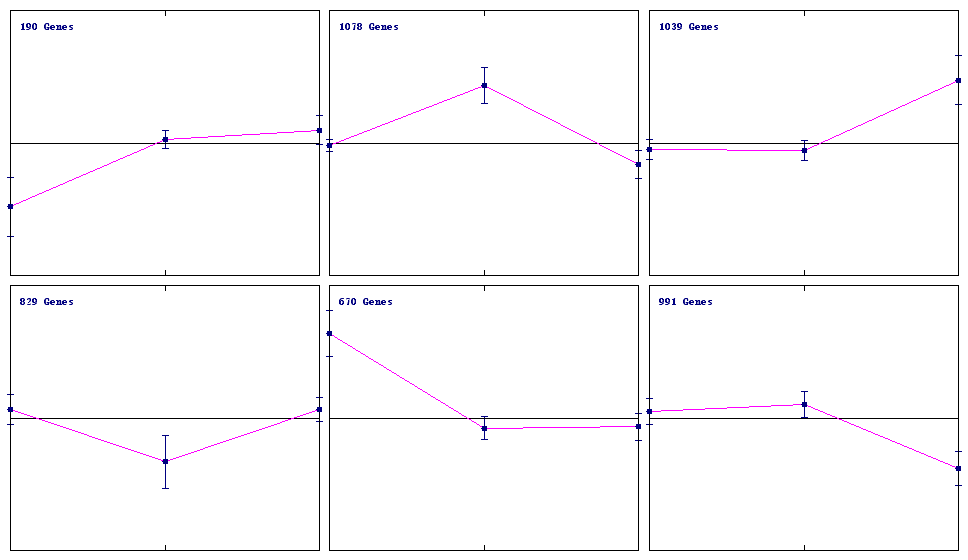


**Figure S3. 7 cluster 1-7 for 2335 DEGs in xylem.**


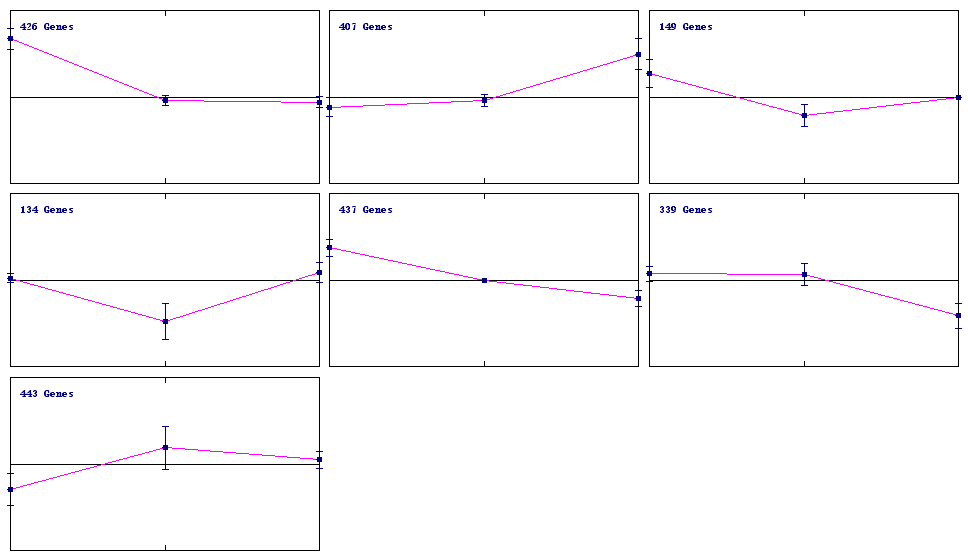


**Figure S4. cluster 1-7 for 3358 DEGs in root.**


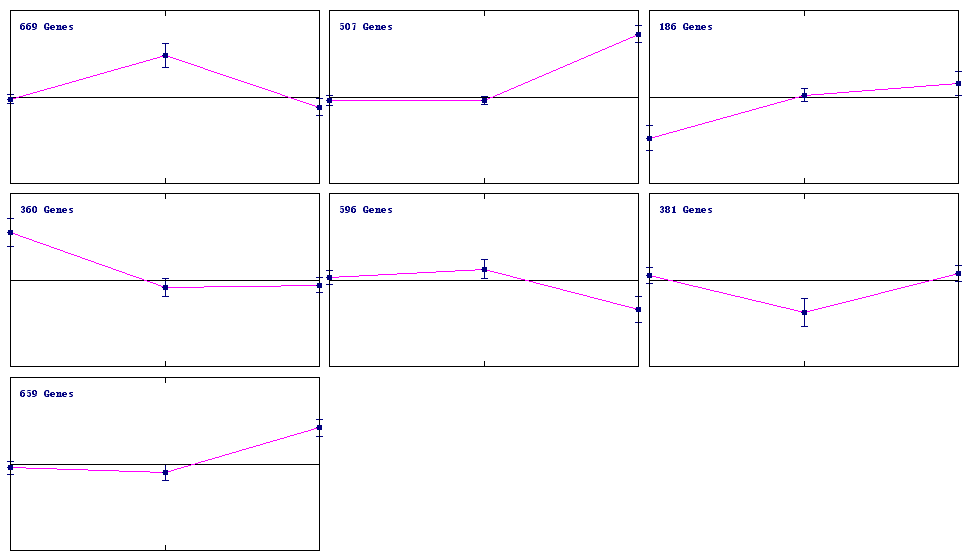

Supplement: Supplementary file 1 [file genes-08-00372-s001.zip › Supplementary File S6.docx]
