# Supplementary figures and images for: Tissue-Specific Transcriptome Analysis Reveals Multiple Responses to Salt Stress in Populus euphratica Seedlings
Source: Genes (Basel). 2017 Dec 8;8(12):372. doi: 10.3390/genes8120372 (PMC5748690; doi:10.3390/genes8120372)

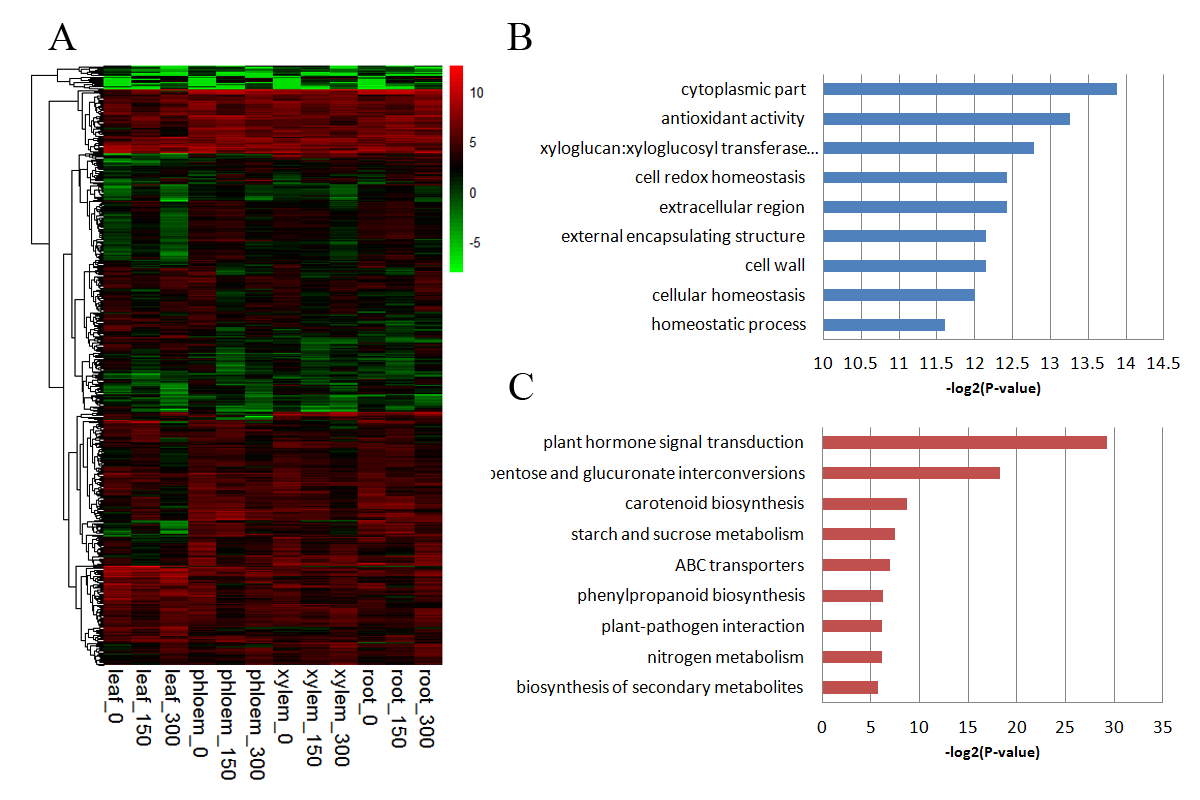

Supplement: Supplementary file 1 [file genes-08-00372-s001.zip › Figure S5.tif]

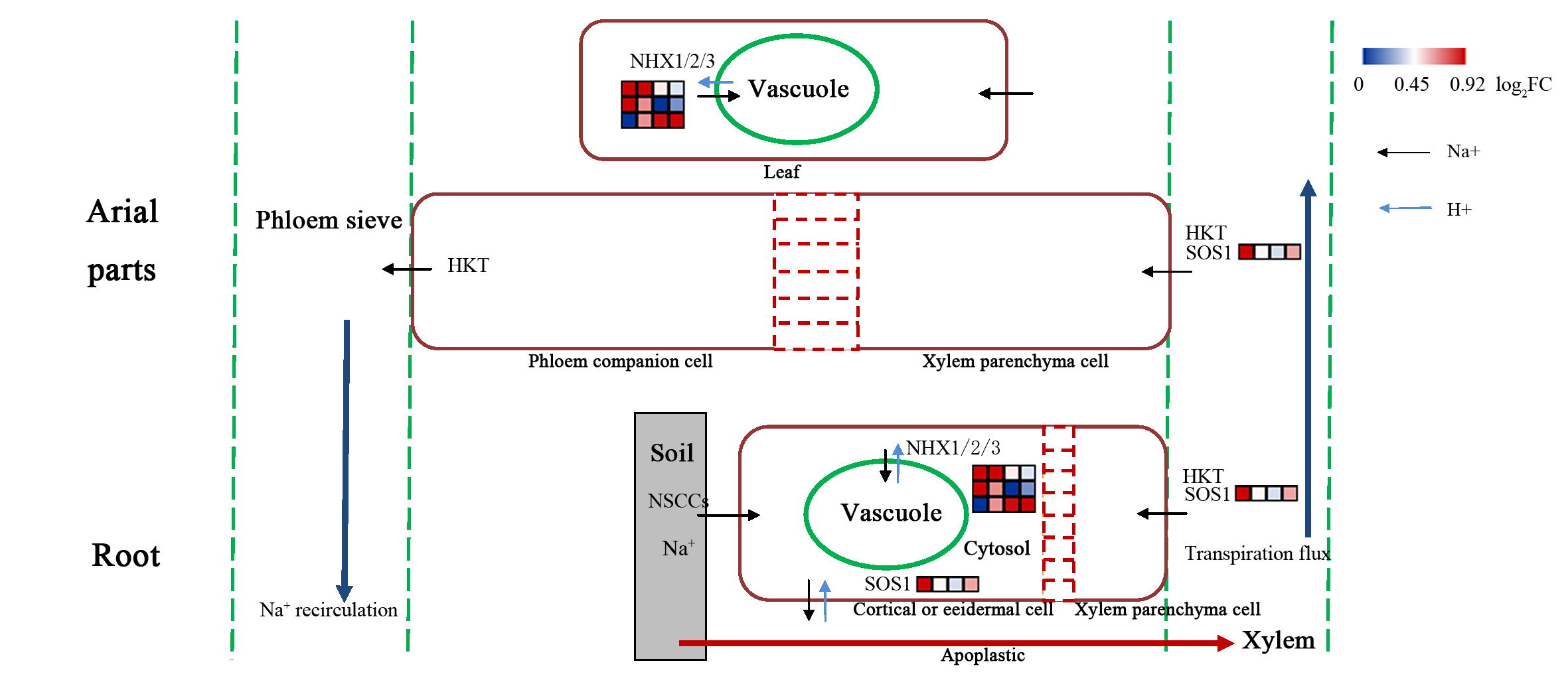

Supplement: Supplementary file 1 [file genes-08-00372-s001.zip › Figure S6.tif]

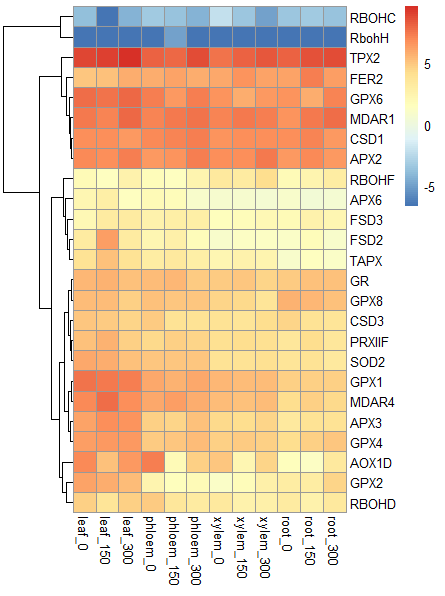

Supplement: Supplementary file 1 [file genes-08-00372-s001.zip › Figure S7.tif]
